# Can electrical impedance tomography predict successful mechanical ventilation weaning in tracheostomized patients? A single-center observational study

**DOI:** 10.36416/1806-3756/e20260049

**Published:** 2026-11-09

**Authors:** Walkyria A Macedo Pinto, Flávio G R Freitas, Fernando J S Ramos, Renato H N Santos, Flávia R Machado

**Affiliations:** 1. Unidade de Terapia Intensiva, Hospital São Paulo, Escola Paulista de Medicina, Universidade Federal de São Paulo, São Paulo (SP) Brasil; 2. Programa de Pós-Graduação em Medicina Translacional, Escola Paulista de Medicina, Universidade Federal de São Paulo, São Paulo (SP) Brasil.

**Keywords:** Respiration, artificial, Ventilator weaning, Electrical impedance, Tracheostomy

## Abstract

**Objective:**

To evaluate whether ventilation variables monitored by electrical impedance tomography (EIT) behaved differently between tracheostomized patients who progressed successfully or failed within 6 h after withdrawal of invasive mechanical ventilation (IMV), as well as to improve the accuracy of failure prediction models.

**Methods:**

This was a single-center prospective observational study including tracheostomized adults who had been on IMV for more than seven days and who were clinically ready for discontinuation. Clinical and EIT variables-end-expiratory lung impedance, impedance variation, center of ventilation, heterogeneity index, and ventilation distribution-were collected. The primary outcome measure was IMV weaning failure within 6 h, defined as the need to resume IMV for respiratory distress. Associations between EIT variables and failure were tested by using multivariate logistic regression and ROC curves to assess predictive accuracy.

**Results:**

Fifty-nine patients were analyzed; 43 (72.9%) were successfully weaned from IMV, and 16 (27.1%) failed. In the region of interest H1 (ventral lung region), end-expiratory lung impedance decreased more markedly among successfully weaned patients (p = 0.03). Respiratory rate behavior differed between the groups over time (interaction p = 0.01), whereas other EIT variables showed no group-time interaction. In the multivariate analysis, BMI and 24-hour fluid balance were independently associated with failure. Adding EIT variables to clinical models did not improve predictive accuracy.

**Conclusions:**

The EIT variables evaluated in our study did not behave differently between tracheostomized patients who were successfully weaned from IMV and those who were not. The addition of EIT variables did not improve the accuracy of failure prediction models.

## INTRODUCTION

Weaning from invasive mechanical ventilation (IMV) is a complex process influenced by multiple physiological and clinical factors, and failure remains frequent even when standard readiness criteria are fulfilled.^1^ ^-^ ^6^ In tracheostomized patients, weaning is particularly challenging because prolonged IMV, respiratory muscle weakness, and multiple comorbidities are common.^7^ ^-^ ^10^ In this context, clinicians often face the dilemma of avoiding respiratory muscle fatigue and unnecessary prolongation of ventilatory support. Decisions are usually guided by clinical examination and global physiological parameters, and the ease of reinstituting IMV in tracheostomized patients may lead to conservative strategies and delayed recovery of ventilatory autonomy.^11^ ^-^ ^14^

Electrical impedance tomography (EIT) is a noninvasive bedside imaging technique that allows real-time assessment of regional ventilation and lung aeration.^15^ ^-^ ^19^ Previous studies of patients undergoing IMV have suggested that changes in EIT-derived variables are associated with weaning failure, including a reduction in end-expiratory lung impedance (EELZ), reflecting loss of lung aeration, and an increase in heterogeneity index (HI), indicating greater ventilation inhomogeneity. However, evidence in tracheostomized patients remains limited.^20^ ^-^ ^26^ Therefore, the objective of the present study was to evaluate whether early changes in EIT variables differed between tracheostomized patients who successfully discontinued IMV and those who failed shortly after discontinuation. 

## METHODS

### Study design

This was a single-center prospective observational study conducted in four public university hospital ICUs in Brazil. The study was conducted between April of 2019 and April of 2021, being interrupted between February of 2020 and August of 2020 because of pragmatic issues related to the COVID-19 pandemic. The study was approved by the Federal University of São Paulo Research Ethics Committee (Protocol no. 03257518.5.0000.5505), and written informed consent was obtained from all participants or their legal representatives prior to enrollment. The study adhered to the Strengthening the Reporting of Observational Studies in Epidemiology guidelines in its design and reporting.^27^

We assessed for eligibility all tracheostomized patients > 18 years of age undergoing IMV for more than 7 days at their first weaning attempt. Patients were included if they were hemodynamically stable-defined as an HR of 70-120 bpm and a mean arterial pressure > 65 mmHg-and were considered by the ICU team to be ready for weaning from IMV. According to our institutional protocol, tracheostomized patients are ready for weaning from IMV if they are on pressure support ventilation of 7 cmH_2_O or less, with a PEEP of 5 cmH_2_O, minute ventilation < 10 L/min, FiO_2_ ≤ 0.4, and SpO_2_ > 90%. Exclusion criteria included pregnant and breastfeeding women; individuals with cardiac pacemakers or other implanted electronic devices; wounds, drains, burns, or sensitive skin precluding the use of an EIT belt; signs of respiratory distress (paradoxical thoracoabdominal movement, nasal flaring, and use of accessory muscles); use of vasoactive drugs exceeding 0.1 µg/kg/min; and EIT recordings of poor technical quality. 

### Outcomes

The primary endpoint was failure of IMV discontinuation within 6 h, defined as the need to resume IMV because of respiratory issues. This time frame was chosen in order to assess early ventilatory failure after withdrawal of positive pressure, focusing on changes related to respiratory mechanics and lung aeration while minimizing the influence of nonrespiratory factors. In addition, according to our institutional protocol, all tracheostomized patients are routinely reconnected to IMV overnight on the first day after discontinuation. Therefore, the six-hour window allows evaluation of ventilatory performance before the scheduled return to IMV. As a secondary outcome, we assessed the same variables using failure to wean from IMV within 48 h. 

### Study protocol and measurements

After the decision to discontinue IMV, patients were monitored with EIT (Enlight 1800; Timpel S.A., São Paulo, Brazil). A self-adhesive belt with 32 electrodes was applied transversely around the chest, just below the axilla. After belt placement, patients were placed in a 45° dorsal position. Airway suctioning was routinely performed at this stage, in accordance with our institutional protocol, to ensure patient comfort and optimize EIT signal quality. These precautions were consistently adopted to minimize artifacts that could compromise data acquisition. 

After EIT signal quality was ensured, maximal inspiratory pressure was measured with a pneumotachograph (Philips Respironics, Murrysville, Pennsylvania, USA). Subsequently, patients were reconnected to IMV with the same ventilatory parameters, and a rest period was allowed until the baseline EIT plethysmogram was restored. Subjects were then disconnected from IMV, and new measurements were obtained at predefined intervals (at 1, 3, 5, 30, and 60 min-T1, T3, T5, T30, and T60, respectively). EIT variables were analyzed within 1 h of discontinuation. In line with our objective of detecting early failure, we selected that observation period to maximize signal stability and feasibility, given that critically ill subjects are prone to interventions and mobilization that might affect accuracy. 

We collected data on the following: EELZ; HI; impedance variation (ΔZ); center of ventilation (COV) X and Y; and ventilation distribution (supplementary material, Table S1). EIT variables were assessed globally (as the sum of four regions of interest-ROI); by region (i.e., ventral [ROI1 + ROI2] and dorsal [ROI3 + ROI4]); and individually by ROI (H1, H2, H3, and H4). Considering the proven association between failure to wean from IMV within 6 h and the immediate impact of positive pressure withdrawal on lung aeration, we also analyzed the difference between measurements at T1 and baseline for EIT variables. Our analysis included the difference between T60 and baseline, allowing us to assess changes in lung aeration over time, including a reduction in lung aeration. EIT variables were not available to the bedside clinical team during data acquisition because they were obtained later through offline analysis. Raw EIT data were continuously recorded and processed for offline analysis with the EIT-Main 8.11.2 software (Timpel S.A.) and the LabVIEW platform, version 7.4 (NI, Austin, TX, USA). 

Vital signs were monitored with multiparametric devices: DIXTAL 2020 (DIXTAL Biomédica Indústria e Comércio Ltda., São Paulo, Brazil) and EFFICIA CM10 (Philips Medical Systems, Amsterdam, The Netherlands). Additionally, demographic and clinical data were collected, including the following: age; sex; BMI; severity scores, including the Simplified Acute Physiology Score (SAPS 3) and SOFA at ICU admission and on the study day; and comorbidities, including the Charlson Comorbidity Index, type of ICU admission, and length of IMV. We also collected data on respiratory parameters (maximal inspiratory pressure and the rapid shallow breathing index-RSBI), 24-hour fluid balance (in mL), ICU Mobility Scale scores, ARDS, and number of extubation attempts before tracheostomy. 

At any time after discontinuation of IMV, patients showing signs of failure were reconnected to IMV, with ventilatory parameters being adjusted as needed for respiratory comfort. We defined failure as the presence of any of the following: a drop in SpO_2_ < 90%; RR > 35 breaths/min; HR > 140 bpm; a change in mean arterial pressure outside the 65-130 mmHg range; agitation; excessive sweating; cyanosis; and evidence of high respiratory effort. The criteria for weaning failure were recorded. The study timeline is shown in Figure S1 (supplementary material). 

### Statistical analysis

Because our primary objective was to explore potential trends, patterns, and associations rather than to test specific hypotheses with predefined statistical power and because of the scarcity of comparable studies of tracheostomized patients, we opted for a convenience sample of 60 patients-a sample size comparable to those of previous studies.^20^ ^-^ ^26^ The findings of the present study might inform more comprehensive future research, which might validate and extend the conclusions drawn from our study. 

Data were presented as proportions for categorical variables and as measures of central tendency (medians or means) with their respective variances (percentiles or standard deviations) for continuous variables. Statistical tests, including ANOVA, the Mann-Whitney test, and Fisher’s exact test, were used in order to compare groups and variables. To evaluate our hypothesis that EIT variables would behave differently in patients who failed IMV discontinuation within 6 h when compared with those who succeeded-specifically, a possible reduction in EELZ and ΔZ and an increase in COV X, COV Y, and HI-we used ANOVA for repeated measures. Group comparisons at each time point were performed by using the least squares means method for the six-hour primary outcome analysis and the secondary analysis with 48 h as a cutoff. 

A multivariate logistic regression model was constructed to analyze factors associated with IMV weaning failure. We evaluated potentially relevant baseline clinical variables, including age, BMI, the Charlson Comorbidity Index, SAPS 3, prior duration of IMV, 24-hour fluid balance, and the RSBI. Variables displaying a value of p < 0.10 in the univariate analysis were selected for inclusion in the initial multivariate logistic regression model. Variables with a value of p ≥ 0.10 in the multivariate analysis were subsequently excluded. Variance inflation factors were calculated to confirm the absence of relevant multicollinearity. To avoid overfitting given the sample size constraints, individual EIT-derived variables were subsequently evaluated by adjusting them one by one for these primary clinical predictors. On the basis of their relevance, we included the following EIT variables: global EELZ, global ΔZ, COV X, COV Y, and HI at baseline. We constructed additional models including the difference between T1 and T60 regarding the baseline value for each of these variables. 

Finally, we constructed ROC curves to determine the AUC with 95% CIs, assessing the discriminative performance of the final clinical model and the model incorporating the clinically adjusted, individual EIT parameter that met the significance criteria (p ≤ 0.10). 

Statistical analyses were conducted with the R statistical software, version 4.4.1 (R Foundation for Statistical Computing, Vienna, Austria), for Windows, the significance level being set at a two-tailed alpha of 5%. 

## RESULTS

During the study period, 2,397 patients were admitted to the ICU. Of those, 961 underwent IMV and 117 were tracheostomized. Of those, 67 were eligible for IMV discontinuation, and 61 consented to participate. Two patients were excluded because of poor EIT signal quality, the final sample therefore consisting of 59 patients. Throughout data acquisition, no participant presented with psychomotor agitation or cognitive-behavioral changes that could compromise EIT signal quality or interfere with the measurements. Of the 59 patients analyzed in the present study, 43 (72.88%) were successfully weaned within 6 h of IMV discontinuation, whereas 16 (27.12%) failed (Figure 1). The reasons for resuming IMV were signs of respiratory fatigue, paradoxical breathing pattern, bronchospasm, and respiratory distress. One patient developed atrial fibrillation, which resolved immediately after reinitiation of IMV. 

**Figure 1:**
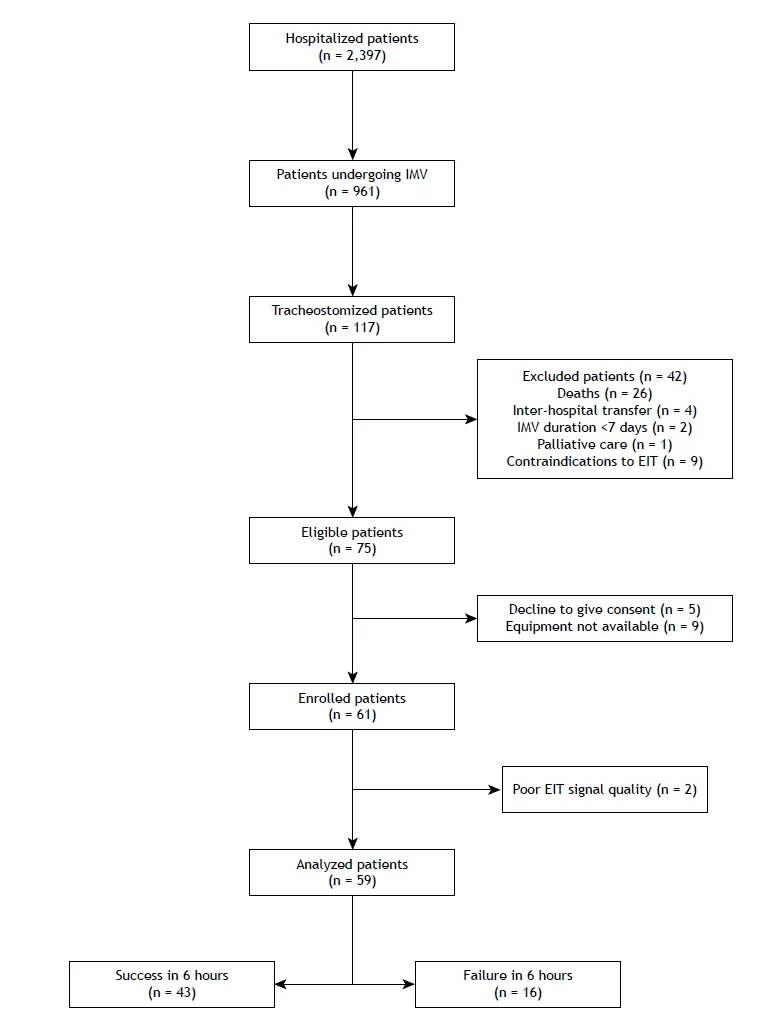
Study flowchart. IMV: invasive mechanical ventilation; EIT: electrical impedance tomography.

The baseline characteristics of the patients are summarized in Table 1. Those who failed weaning had a higher BMI; a higher prevalence of COPD and obesity; and a more positive fluid balance in the 24 hours preceding IMV discontinuation. Although those patients had longer ICU and hospital stays, no significant difference in mortality was observed. In contrast, successfully weaned patients had a higher prevalence of neurological diseases and a higher Charlson Comorbidity Index. 

**Table 1:** Characteristics of the study population, by successful or failed weaning from invasive mechanical ventilation at 6 h.^a^

Variables	Overall (n = 59)	Success (n = 43)	Failure (n = 16)	p
Female sex	36 (61%)	28 (65.1%)	8 (50%)	0.37
Age, years	53.2 ± 16	53.9 ± 16.5	51.6 ± 15.1	0.63
BMI, kg/m^2^	25.6 ± 5.5	24.5 ± 4.5	28.5 ± 6.8	0.01
Severity scores				
SAPS 3	64.3 ± 16.2	66.5 ± 15.8	58.5 ± 16.2	0.09
SOFA (at ICU admission)	7.2 ± 3.5	7.3 ± 3.6	6.9 ± 3.3	0.69
SOFA (on study day)	4 [3-6]	4 [3-6]	4.5 [3-8]	0.75
Type of admission				0.71
Clinical	42 (71.2%)	29 (67.4%)	13 (81.2%)	
Surgical	5 (8.5%)	4 (9.3%)	1 (6.2%)	
Emergency surgery	12 (20.3%)	10 (23.3%)	2 (12.5%)	
Category admission				0.97
Emergency room	41 (69.5%)	30 (69.8%)	11 (68.8%)	
Ward	9 (15.3%)	6 (14%)	3 (18.8%)	
Operating room	7 (11.9%)	5 (11.7%)	2 (12.4%)	
Another hospital	2 (3.4%)	2 (4.7%)	0 (0%)	
Reasons for ICU admission				0.54
Respiratory failure	27 (45.8%)	17 (39.5%)	10 (62.5%)	
COVID-19	18 (30.5%)	12 (27.9%)	6 (37.5%)	
Postoperative period	17 (28.8%)	15 (34.9%)	2 (12.4%)	
Neurosurgical postoperative period	11 (18.6%)	10 (23.3%)	1 (6.2%)	
Neurological disease	12 (20.3%)	9 (20.9%)	3 (18.8%)	
Neuromuscular disease	2 (3.4%)	2 (4.7%)	0 (0%)	
Cardiorespiratory arrest	2 (3.4%)	1 (2.3%)	1 (6.2%)	
Sepsis	1 (1.7%)	1 (2.3%)	0 (0%)	
Comorbidities				
Hypertension	37 (62.7%)	29 (67.4%)	8 (50%)	0.24
Neurological disease	30 (50.8%)	26 (60.5%)	4 (25%)	0.02
Diabetes mellitus	25 (42.4%)	21 (48.8%)	4 (25%)	0.14
Obesity	11 (18.6%)	5 (11.6%)	6 (37.5%)	0.051
Chronic lung disease	8 (13.6%)	2 (4.7%)	6 (37.5%)	0.003
Heart failure	1 (1.7%)	1 (2.3%)	0 (0%)	0.99
Charlson Comorbidity Index	2 [0-3]	2 [0.5-3]	1 [0-1.2]	0.031
Respiratory dysfunction				
Previous ARDS	39 (66.1%)	27 (62.8%)	12 (75%)	0.53
Prior duration of IMV, days	21 [15.5-27.5]	21 [15-27]	20.5 [18.5-30]	0.60
Prior discontinuation attempts				
0	37 (62.7%)	27 (62.8%)	10 (62.5%)	0.75
1	14 (23.7%)	11 (25.6%)	3 (18.8%)	
2	6 (10.2%)	4 (9.3%)	2 (12.5%)	
3	2 (3.4%)	1 (2.3%)	1 (6.2%)	
MIP, cmH_2_O	−35 [−47 to −25.2]	−31 [−45 to −25]	−40.1 [−49.2 to −32]	0.37
RSBI	49.6 [35.8-70]	49 [36.3-70]	57.5 [31.2-70]	0.84
24-hour fluid balance, mL	858 [60.5-1,364]	800 [14-1,185]	1,103 [626-1,933.2]	0.051
ICU Mobility Scale score				0.83
0	34 (57.6%)	24 (55.8%)	10 (29.4%)	
1-2	13 (22%)	9 (20.9%)	4 (25%)	
3	5 (8.5%)	4 (9.3%)	1 (6.3%)	
4-6	7 (11.9%)	6 (14%)	1 (6.3%)	
Vasopressor use				
Noradrenaline	9 (15.3%)	7 (16.3%)	2 (12.5%)	0.99
Dobutamine	1 (1.7%)	1 (2.3%)	0 (0%)	0.99
Sodium nitroprusside	1(1.7%)	0 (0%)	1 (6.2%)	0.27
Clinical outcomes				
Length of ICU stay, days	35 [26.5-57.5]	32 [25.5-50.5]	57.5[34.8-66.5]	0.01
Length of hospital stay, days	69 [42.5-95]	53[39.5-85]	85.5 [66-141]	0.02
ICU mortality	15 (25.4%)	10 (23.3%)	5 (31.3%)	0.53
In-hospital mortality	19 (32.2%)	14 (32.5%)	5 (31.3%)	0.92

SAPS 3: Simplified Acute Physiology Score; IMV: invasive mechanical ventilation; and RSBI: rapid shallow breathing index. ^a^Values expressed as n (%), mean ± SD, or median [IQR]. Fisher’s exact test. ANOVA and the Wilcoxon test, as appropriate.

Regarding EIT-derived variables, both groups exhibited a decrease in global EELZ at T1 and throughout the first hour after IMV discontinuation (p < 0.001). A significant difference was observed between the success and failure groups (p = 0.04), although CIs of central tendency measures overlapped. No interaction was found between the reduction in EELZ over time and the success or failure group (p = 0.16; Figure 2). For the remaining EIT variables, no significant differences were observed between the groups (supplementary material, Table S2). When we analyzed the differences between T1 and baseline, as well as the differences between T60 and baseline, we found no significant differences. 

**Figure 2:**
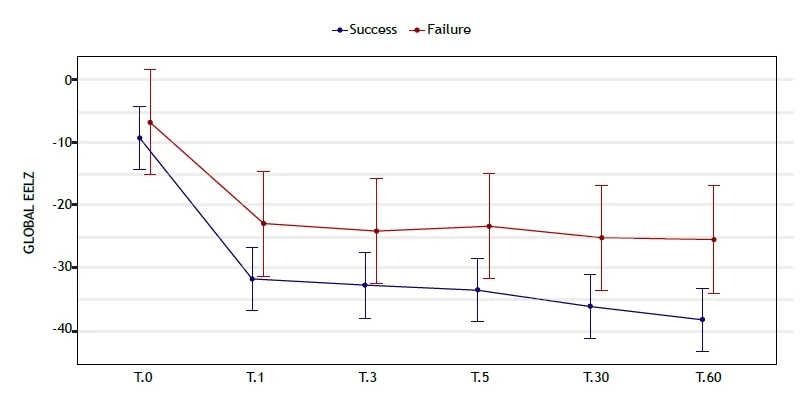
Variation in global end-expiratory lung impedance (EELZ) according to the six-hour outcome after mechanical ventilation discontinuation. Two-way ANOVA showed a significant effect of group (p = 0.04) and time (p < 0.001), with no significant group-time interaction (p = 0.16).

Regional ventilation analysis demonstrated a reduction in EELZ in the ventral lung region (H1 + H2) during the first minute and over the first hour following IMV discontinuation in both groups (p < 0.001), with no interaction between group and time (p = 0.16). In ROI H1, a significant interaction between group and time was observed (p = 0.03), with a more pronounced drop in EELZ in the patients who were successfully weaned (Figure 3). No other ventral regional differences were identified between the groups. In the dorsal lung region (H3 + H4), we also observed a drop in EELZ and ΔZ in the first minute and over the first hour in both groups, with no interaction. Regarding the distribution of ventilation, we observed an increase in ventilation in the dorsal region, and a consequent drop in the ventral region, in both groups, with no significant differences between the groups (supplementary material, Tables S3 and S4). 

**Figure 3:**
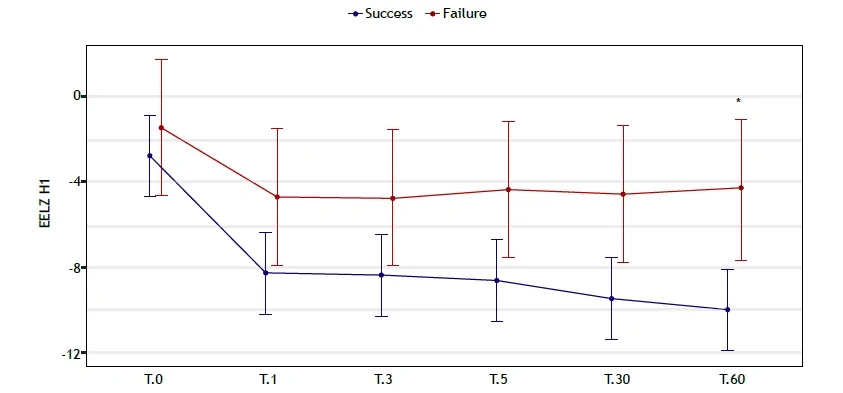
Variation of end-expiratory lung impedance (EELZ) in region of interest H1 according to the six-hour outcome after invasive mechanical ventilation discontinuation. Repeated-measures ANOVA showed significant effects of group (p = 0.02) and time (p < 0.001), with a significant group-time interaction (p = 0.03). *p < 0.05 for between-group comparison at the indicated time point.*

In parallel with EIT measurements, tidal volume and minute ventilation decreased significantly after IMV discontinuation, with no differences between the groups. A significant interaction between group and time was observed for RR (p = 0.01), with a reduction among successfully weaned patients and an increase in those who failed. The RSBI varied significantly over time (p < 0.001), with no differences between the groups (p = 0.31; supplementary material, Table S5). 

When the observation period was extended to 48 h, 9 additional patients failed to wean after the initial six-hour period, totaling 25 patients (42.4%). Consistently with our primary results, the first-minute EIT-derived variables changed over time, with progressive reductions in EELZ and increased ventilation heterogeneity. However, no differences were found between the success and failure groups for global EIT parameters. Ventilation distribution indices and tidal impedance variation also remained similar between the groups. In the regional analysis, a difference was observed in ROI H1, with a greater reduction in EELZ in the success group, although this was not consistent across other regions. Respiratory rate showed a significant time-by-group interaction, indicating different temporal patterns between the groups, similar to what was observed in the six-hour analysis. Detailed results are provided in the supplementary material (Tables S6 to S9). 

In our models to determine the clinical variables associated with failure of IMV discontinuation, the baseline clinical variables were evaluated by univariate logistic regression analysis. Then, selected variables were included in the initial multivariate model (supplementary material, Tables S10 to S12). Only BMI (OR = 1.16; 95% CI, 1.03-1.31; p = 0.02) and fluid balance (OR = 1.05; 95% CI, 0.99-1.11; p = 0.08) remained in the model (supplementary material, Table S12), with an AUC of 0.75 (95% CI, 0.60-0.90), a sensitivity of 81.2%, and a specificity of 69.8% (Figure 4). Subsequently, individual EIT-derived variables were incorporated into the clinical model in a stepwise manner. However, none of those variables were found to be associated with IMV discontinuation failure (supplementary material, Table S13). Although baseline global EELZ met the predefined significance criterion (p ≤ 0.10), its inclusion did not improve model discrimination (AUC = 0.77; 95% CI, 0.62-0.91; sensitivity 87.5%; specificity 74.4%; Figure 5). 

**Figure 4:**
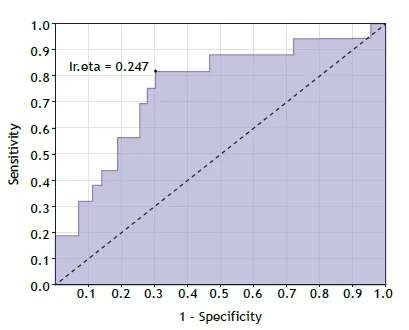
ROC curve after invasive mechanical ventilation discontinuation-final clinical model. Variables included in the model were as follows: BMI (OR = 1.16; 95% CI, 1.03-1.31; p = 0.02); 24-hour fluid balance increment = 100 mL (OR = 1.05; 95% CI, 0.99-1.11; p = 0.08); sensitivity = 0.812; specificity = 0.698; AUC = 0.75 (95% CI, 0.6-0.90).

**Figure 5:**
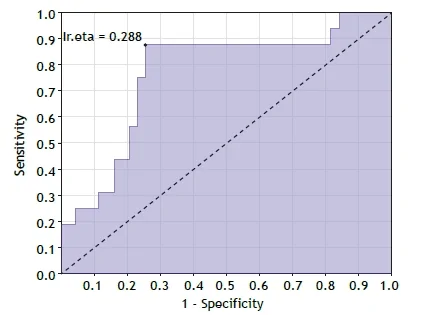
ROC curve after invasive mechanical ventilation discontinuation-final clinical model with the addition of end-expiratory lung impedance. Variables included in the model were as follows: BMI (OR = 1.18; 95% CI, 1.04-1.35; p = 0.01); 24-hour fluid balance increment = 100 mL (OR = 1.06; 95% CI, 1.00-1.13; p = 0.04); end-expiratory lung impedance (OR = 1.09; 95% CI, 0.98-1.21; p = 0.10); sensitivity = 0.87; specificity = 0.744; AUC = 0.77 (95% CI, 0.62-0.91).

## DISCUSSION

In our prospective study of tracheostomized critically ill patients undergoing their first attempt at IMV discontinuation, we observed significant changes in EIT variables within the first 60 minutes after discontinuation. However, those changes were similar between patients who succeeded and those who failed, except for a more pronounced decrease in EELZ in the ROI H1 in the ventral region among those who were successfully weaned. The inclusion of EIT variables, including EELZ, in multivariate models did not improve the prediction of early IMV discontinuation failure. However, given the limited sample size, we might not be adequately powered to detect associations. Therefore, our results should be considered exploratory and used as a “proof-of-concept.” 

Our results are different from those reported in previous studies identifying EIT parameters as predictors of success.^23^ ^-^ ^24^ This might be attributed to the specific characteristics of our cohort. Tracheostomized patients undergoing prolonged weaning may develop compensatory respiratory patterns and exhibit different thoracoabdominal mechanics in comparison with the acutely intubated patients typically found in the literature. 

The global reduction in EELZ observed after IMV discontinuation in the present study is consistent with previous reports and likely reflects loss of lung recruitment, alveolar collapse, and regional ventilation instability following removal of positive pressure.^20^ ^-^ ^22^ Thus, we hypothesized that patients failing the weaning process would exhibit a more significant decrease in EELZ and an increase in HI, signaling alveolar collapse and regional ventilation instability. Indeed, previous studies have shown that a decline in EELZ during a T-piece trial could predict spontaneous breathing trial failure in patients requiring prolonged weaning.^20^ Other studies have shown that greater heterogeneity and loss of aeration, as monitored by EIT, are associated with weaning failure in intubated patients.^21^ ^,^ ^25^

In contrast to the aforementioned findings, our results in tracheostomized patients revealed that the reduction in EELZ was more marked in those who were successfully weaned, specifically in ROI H1. A possible explanation is that patients who failed had lower lung compliance or higher airway resistance, resulting in less variation in EELZ relative to baseline. The localized pattern observed in ROI H1 may also reflect regional differences in compliance or ventilation redistribution, possibly related to pendelluft phenomena during spontaneous breathing. Similar regional impedance patterns have been previously described using EIT in spontaneously breathing patients, supporting this physiological interpretation.^28^ However, these mechanisms were not directly assessed in the present study and should be considered as hypothesis generation to be explored in future studies. 

As expected, BMI and fluid balance were independently associated with IMV discontinuation failure, reinforcing the multifactorial nature of the weaning process. EIT-derived variables, which primarily reflect respiratory mechanics, may be insufficient to capture the complexity of factors involved in ventilatory autonomy. Although EELZ showed potential physiological relevance, its clinical applicability as a standalone decision-making tool remains limited. 

Our findings should also be interpreted in light of current standards such as the Wind study classification,^6^ which define weaning outcomes based over a longer period. The six-hour observation window was selected to focus on early ventilatory failure in order to evaluate physiological changes immediately after withdrawal of ventilatory support potentially related to respiratory mechanics and lung aeration, minimizing the influence of nonrespiratory factors such as infection, metabolic disturbances, and hemodynamic instability. We believe that tracheostomized patients show specific clinical features that justify this approach. In this population, reinitiation of IMV is technically simple and can be rapidly implemented, which may lower the threshold for reconnecting patients even in the absence of true or sustained weaning failure. Thus, detecting early changes in EIT may provide clinically actionable information, particularly by increasing confidence in maintaining spontaneous ventilation when early adaptation is adequate. 

The present study has strengths, including prospective data collection and the use of continuous bedside imaging. However, limitations such as the single-center design, small sample size, and heterogeneity of the study population may limit generalizability. Our primary outcome, i.e., early EIT-derived changes, may not fully reflect the subsequent clinical course, and unmeasured factors may have contributed to later failure; therefore, our findings should be interpreted with caution. Further studies are warranted to better define the role of EIT in supporting clinical decision-making during IMV discontinuation in tracheostomized patients. 

In our study population and design, the assessed EIT variables did not differ between those who were successfully weaned from IMV and those who failed. The addition of EIT variables did not improve the accuracy of models predicting IMV discontinuation failure. 
